# Coupled oscillator mechanism extends the amplifying effect of outer hair cells to high frequencies

**DOI:** 10.1038/s41598-025-33079-3

**Published:** 2025-12-22

**Authors:** Kuni H. Iwasa

**Affiliations:** 1 NIDCD, National Institutes of Health, Bethesda, MD 20892 USA; 2 National Institute on Deafness and Other Communication Disorders, National Institutes of Health, Bethesda, MD 20892, USA

**Keywords:** Hearing, Sensitivity, Coupling oscillators, Mechanics, Biophysics, Engineering, Neuroscience, Physics, Physiology

## Abstract

Outer hair cells (OHCs) are essential for the sensitivity and frequency specificity of the mammalian ear. To perform this function, OHCs need to amplify the motion of the basilar membrane, which is much stiffer than themselves. OHCs must overcome this impedance mismatch for their amplifying function particularly at high frequencies, where the mismatch is the largest. This issue could be solved by the existence of multiple modes of motion. Here, systems of two coupled oscillators are examined as the simplest of such cases. It is found that some of these model systems indeed make OHCs function as an effective amplifier by overcoming the impedance mismatch. This result suggests that the elaborate structure of the organ of Corti, which can support multiple modes of motion, is a key to the high frequency performance of the mammalian ear.

**Supplementary Information:**

The online version contains supplementary material available at 10.1038/s41598-025-33079-3.

## Introduction

The mammalian hearing range may extend up to 100 kHz or beyond^1^, depending on the species, quite remarkable for a biological system. Such function depends on the unique “electromotile” machinery in outer hair cells (OHCs)^2^ and also special mechanisms that make this motility effective.

Electromotility^3^ is based on the piezoelectricity of the lateral wall^4^. As such, the response to direct mechanical stimulation to the cell body is intrinsically fast. However, this direct path is much less sensitive than an indirect path via the mechanoreceptors in the hair bundle, mediated by the membrane potential^5,6^. This path is, however, subjected to attenuation by the low-pass ($\sim$ kHz) nature of their intrinsic electric circuit, due to the capacitance of the cell membrane. In a system with resonance, this attenuation can be absent near the resonance frequency, where the membrane capacitance diminishes^7,8^.

Another hurdle is that OHCs, which are rather soft, need to drive the much stiffer basilar membrane (BM), which is essential for frequency selectivity. This mismatch reduces the transmission of energy. If an OHC and the basilar membrane form a single oscillator, the upper limit of the effectiveness is $\sim$10 kHz^5,8^, not high enough for covering the auditory range of mammalian hearing.

This energy transmission could be improved if the OHCs and the BM are elements of separate oscillators and energy transfer between them is effective. Multiple modes of motion, which place OHCs and the BM in separate oscillators, are reported by recent experimental reports, using optical coherence tomography (OCT)^9–13^. In addition, there are theoretical models, which place OHCs between two inertial masses to explain the tuning curve of the ear^14–18^. Even though the neuronal output of the cochlea is better correlated with the movement of the reticular lamina^19,20^, BM motion is essential, being the basis for the tonotopic map of the cochlea^21^.

All these studies, to my knowledge, do not address the effectiveness of energy transfer between oscillators at high frequencies, where impedance mismatch is the most pronounced^8^. The objective of the present study is to examine the effectiveness of energy transfer between the oscillators in the simplest cases of coupled oscillators. The models used retain the essential features of the cochlea together with the properties of OHCs determined from isolated cell preparations.

More specifically, we examine a set of two oscillators, heavy and light, coupled with either elastic or viscous element. First, a single mode oscillator with an OHC is described to illustrate the effect of impedance mismatch. Then systems of two coupled oscillators are examined. The light oscillator (LO) incorporates an OHC and the heavy one (HO) includes the BM. Coupling is either elastic or viscous. The OHC is stimulated by either the motion of HO or that of LO.

The present treatment focuses on local energy balance to address the upper bound of the auditory frequency. This condition is realized at locations, where energy influx and out-flux along the lateral direction are equal. Since mammalian ear has traveling waves^21^, and with them energy flow exists^22–24^, local models are simplifications. Nonethless it has been shown that local energy balance is satisfied at any location, where the traveling wave of a given frequency stops^23^. The results obtained from the present study are, therefore, necessary conditions.

## Results

To examine the performance of the coupled oscillator models at high frequencies, four cases of coupled oscillator models were formulated and compared with a single oscillator model, which consists of an OHC, a damper, and an elastic element with the stiffness of the BM (Fig. 5 in Methods).

A coupled oscillator system consists of two harmonic oscillators, light and heavy, which a coupling element. The light oscillator (LO) consists of a soft OHC and a small elastic load and small mass. The heavy oscillator (HO), which incorporates the stiff BM, a damper, and large mass. We assume, in general, that the two oscillators are coupled by either an elastic element or a damper for simplicity. An external force is applied to HO (Fig. 6 in Methods).

Numerical analysis is performed using the parameter values (See Methods) that match the mechanical resonance frequency near 40 kHz, which is the auditory limit of guinea pigs, from which the values of OHC parameters are determined.

### Single mode oscillator

Let us start from the performance of the single mode oscillator before examining coupled oscillator models because this model illustrates the effect of impedance mismatch and serves as a control.

**Fig. 1:**
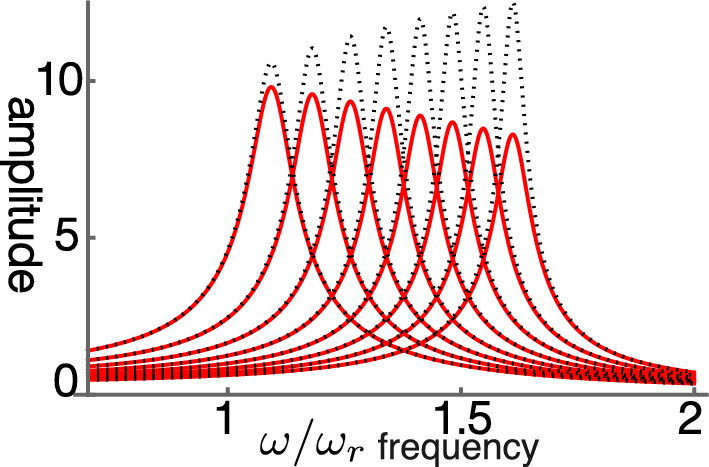
Amplitude of single mode oscillator plotted against frequency (normalized to $\omega _r$). Peak values are re-plotted in Fig. 2A. The ratio of the load stiffness to that of OHC is 10 (stiffness ratio), assuming the BM is the elastic load (red). The result of elastic ratio of 5 (dotted) is shown for comparison. The operating point variable $\gamma$ (see the text for definition) runs from 0.03 (left) to 0.24 with increment of 0.03. The unit of the ordinate axis is $f(=F/k)$, proportional to the amplitude of external force.

**Fig. 2:**
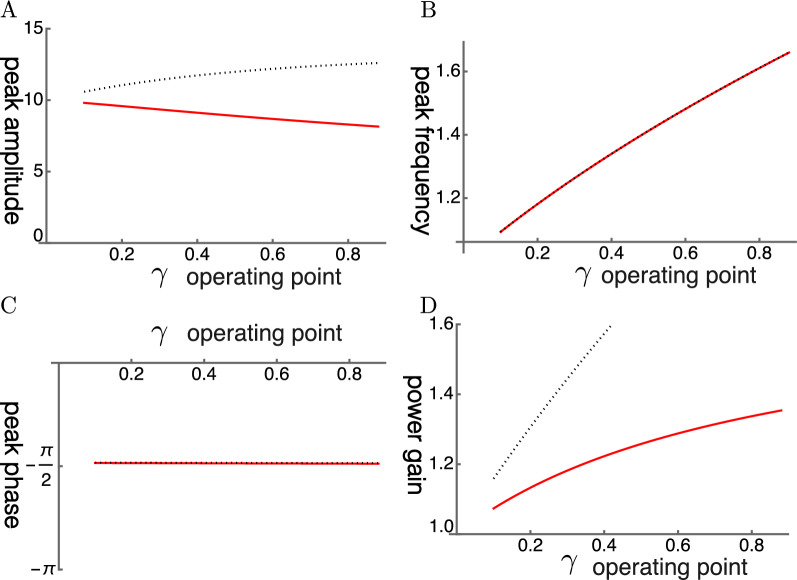
(**A**) Peak amplitude, (**B**) Peak frequency (normalized to the mechanical resonance frequency), (**C**) peak phase, and (**D**) Power gain (ratio of power output to power input) are respectively plotted against $\gamma$, the operating point variable. The stiffness ratio $k/k_o$ is 10 (red). Stiffness ratio of 5 (dotted) is shown for comparison. The peak phase is close to $-\pi /2$, consistent with resonance.

The steady state amplitude is obtained by solving Eq. 5. The amplitude is normalized by the magnitude of force input $\overline{f}$ (Fig. 1). With increasing operating point variable $\gamma$, the shorthand for the normalized sensitivity of the motile response to voltage changes of the OHC, the peak amplitude shifts to higher frequencies independent of the elastic load (Fig. 2B). The peak height, however, does not increase with the voltage sensitivity $\gamma$ because of the large external elastic load $k/k_o=10$, which is represented by the ratio of the stiffness *k* of the external load to the cell stiffness $k_o$. Only if the stiffness ratio is reduced to 5 or lower, does the peak height increase (Fig. 2A).

The peak frequency is plotted as the ratio to the mechanical resonance frequency. The peak frequency shifts away from the mechanical resonance frequency with increasing $\gamma$, the operating point variable (Fig. 2 B). Such a shift has the effect of reducing the peak height. While with a lower stiffness ratio this frequency shift is the same (Fig. 2 B), the peak amplitude increases with $\gamma$. The reason for this difference is that the anti-drag factor *A* is larger for a smaller stiffness ratio while the shift factor *B* is unaffected (See Methods).

The phase of the oscillator lags by $\pi /2$ as expected from the maximal amplitude of the damped oscillator (Fig. 2C). That makes power gain similar to the amplitude of oscillation (Fig. 2D). The low power gain is significantly attenuated by input impedance mismatch.

### Coupled oscillators

In the following, we will examine if OHCs can be more effective in coupled oscillators than in the single mode oscillator. The coupled oscillator models have more parameters, which include stiffness ratio *s* and resonance frequency ratio $\omega _1$ of the two oscillators, and coupling parameters (elastic element $k_c$ and viscous element $\eta _c$).

Conditions for large gain in amplitude are sought in all four cases. It turned out that large gains can be obtained in the systems with elastic coupling. In the following, examples are shown of the systems with elastic coupling. These examples are typical ones but they do not necessarily show all shared features of the conditions.

Viscous coupling is also capable of producing amplification even though their gain is smaller elastic coupled coupling. Examples of cases with viscous coupling are shown in y Material.

#### elastically coupled HO-driven cases

The HO-driven mode is interesting because we assume that HO is associated with the dominant drag, which likely stems from the shear in the subtectorial space and this shear should stimulate the hair bundle of the OHC (Fig. 6 A).

**Fig. 3:**
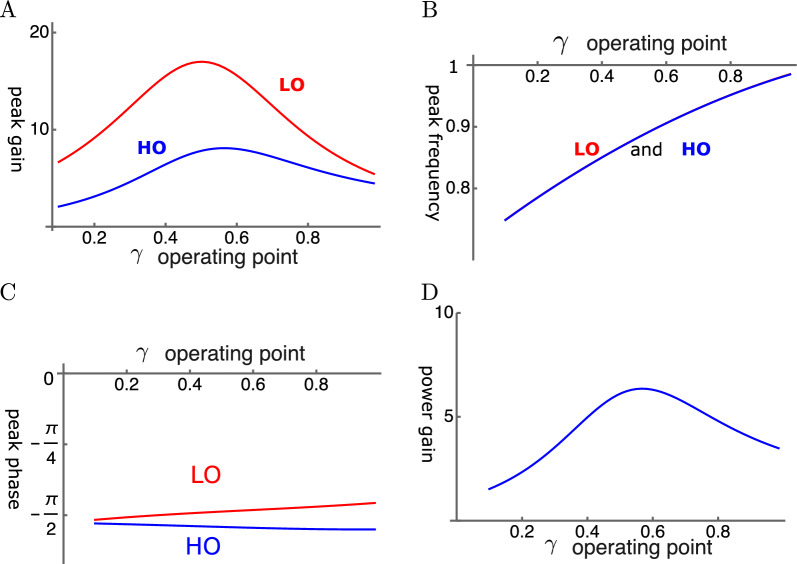
HO-driven elastically coupled oscillators (HOE). (**A**) Amplitude gain over SO mode, (**B**) Peak frequency (normalized to the mechanical resonance frequency), and (**C**) The phase with respect to the external force, of each oscillator are plotted against $\gamma$. LO (red) and HO (blue). (**D**) Power gain. The set of parameter values: $c=k_c/K$=0.2, $\omega _1$=0.29 in Eq. 11. See Table 1 for definitions.

With elastic coupling, it is possible to find conditions, under which the amplitude gain of the LO reaches $\sim$ 20 fold over the single mode oscillator (Fig.  3A). However, the amplitude gains reach their maximal values before $\gamma$, the operating point parameter, reaches unity.

**Table 1:** Parameter definitions.

Symbol	Definition	Parameter	Definition
$P_0$	Motor conformation$^\star$	$\beta$	$1/(k_BT)$
*a*	Prestin displacement	$\gamma$	$4 P_0(1-P_0)$
$\omega _r$	Resonance frequency	$u_a$	$\beta a^2k_oN/4$
*q*	Prestin charge	$u_q$	$\beta q^2 N/(4C_0)$
*N*	Number of prestin	*u*	$\beta a q N/(4\omega _rC_0)$
$C_0$	Cell capacitance	$\omega _\eta$	$K/\eta$
$i_0$	Resting current	$\omega _1$	Resonance frequency ratio
$k_o$	Cell stiffness	$\hat{r}$	Relative change of HB resistance
$k_e$	Parallel elastic load	$\sigma$	Membrane conductance
$k_c$	Coupling spring	$f_B$	External force (amplitude)

$k_B$ is Boltzmann’s constant, *T* the temperature. $^\star$ The motile activity of OHCs is described by a two state model. $P_0$ is the fraction of one of the two states in a single cell at the operating point. In the absence of elastic coupling $k_c=0$

The peak frequency is about the same for the two oscillators and increases with the operating point variable (Fig. 3B). This behavior is similar to that of SO. However, opposite to SO, the shift brings the peak frequency closer to the mechanical resonance frequency, contributing to the peak gain.

The phase of LO is ahead of HO, consistent with the amplifying role of LO. However, the phase difference is rather small (Fig.  3C). The power gain coincides with the amplitude maximum of HO and reaches about 6 at the peak (Fig.  3D).

#### Elastically coupled LO-driven cases

**Fig. 4:**
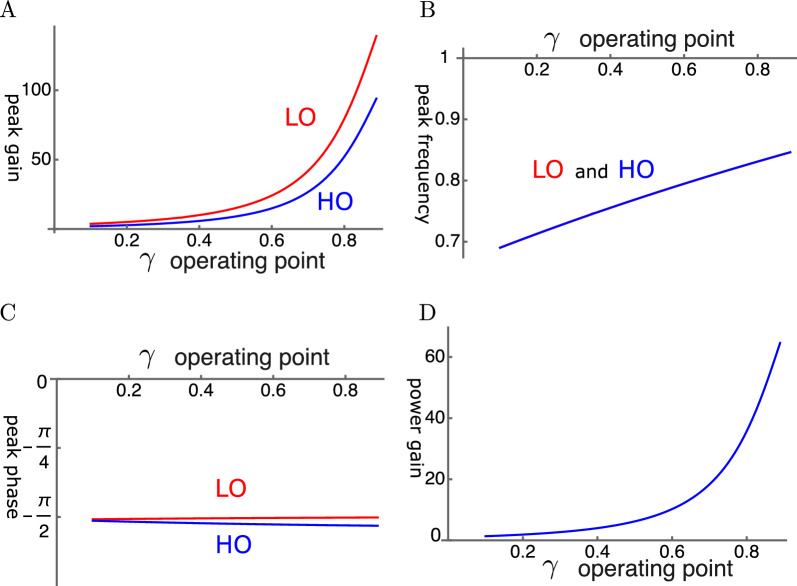
An example of LO-driven elastically coupled oscillators (LOE). (**A**) Amplitude gain over SO mode, (**B**) Peak frequency (normalized to the mechanical resonance frequency), and (**C**) The phase with respect to the external force, of each oscillator, is plotted against $\gamma$. LO (red) and HO (blue). (**D**) Power gain. The set of parameter values: $c=k_c/K$=0.6, $\omega _1$=0.18 in Eq. 13. See Table 1 for definitions.

Here we assume that the OHC is primarily driven by the motion of LO rather than that of HO (Fig. 6 B). With elastic coupling, the amplitude gain can exceed 100 fold for LO and 80 fold for HO (Fig. 4A). The amplitude gain generally increases with the operating point variable $\gamma$ as expected. This increase can be monotonic as in Fig. 4A. However, it can peak before $\gamma$ reaches unity, similar to HO-driven cases, depending on the set of parameter values.

The peak frequencies are similar for both oscillators and increases with $\gamma$ (Fig. 4B). This behavior is similar to that of the Single-mode Oscillator (SO). However, the difference is that the shift brings the peak frequency closer to the mechanical resonance frequency, contributing to the peak gain, similar to a HO-driven case shown in Fig. 3.

The LO is ahead of HO in phase, indicating the amplifying role of the LO even though the difference is rather small (Fig. 4C). The effect of the LO in affecting the amplitude of LO is quite large despite its much smaller mass and stiffness.

The power gain reaches about 60 where the amplitude gain of HO is maximal (Fig. 4D).

#### Interference of coupling

The case of LO-stimulation with elastic coupling (LOE mode) is the most effective in utilizing OHC. It leads to about 60 fold power gain (Fig. 4D) and amplitude gain of about 80 fold for HO (Fig. 4D). The case of HO-stimulation with elastic coupling (HOE mode) may lead to a relatively good amplitude gain for LO, even though it is less effective than LOE. In view of similar phase relationships of the oscillators in those two cases, a superposition of these two stimulation modes could be constructive.

## Discussion

The numerical examination presented here concerns only high frequency performance. At lower frequencies, such as 10 kHz, viscosity coupled cases could produce reasonable gain. However, a high amplifier gain may not be a critical condition at such frequency ranges.

The coupled oscillator models can make OHCs more effective by reducing the constraint of impedance mismatch. However, the performance of coupled oscillator models significantly varies depending on the type of coupling and how OHCs are stimulated.

More specifically, optimal amplification of this system by the OHC is achieved in the case where coupling is elastic and the OHC is stimulated primarily by the oscillation of LO. Under such conditions, the gain is not impeded by a large stiffness mismatch between the OHC and the BM.

Strong coupling is required for making OHCs effective. In the case of elastic coupling, for example, the stiffness of the coupling element must be similar to that of the elastic element of LO. This condition appears to lead to another feature that the resonance frequency of LO is somewhat lower than that of HO. With increased OHC electromotility (increased $\gamma$), the resonance frequency tends to increase, reminiscent of the half-octave shift^25,26^.

Amplitude gain of a coupled oscillator is in general an increasing function of $\gamma$, the operating point as expected. Some of the cases (See Fig. 4A) show monotonic increase with $\gamma$ similar to the single mode oscillator with low elastic load (See Fig. 2A). However, depending on the condition, it can peak before the operating point variable $\gamma$ reaches unity (See Fig. 3A). Such peaking disappears by reducing the value of *B*, which shifts the resonance frequency.

We could speculate that such a behavior could explain a report that lowering prestin density in OHCs by 34% does not have any reduction in the sensitivity of the ear^27^. That is because both *A* and *B* are proportional to *N*, the number of motile units in an OHC (See Table 1). Thus, *N* and $\gamma$ have the same effect on these parameters.

Amplitude gains are very sensitive to small variations of the cochlear parameters. Such a sensitivity may result in uneven power gain. Amplitudes can be smooth functions of the operating point variable $\gamma$ as shown in the figures. They can also show singular dependence on $\gamma$, indicating spontaneous oscillation. These observations could have some bearing on otoacoustic emissions^28^.

The cell body of OHCs is held by the reticular lamina at the apical end, forming tight junctions, and by Deiters’ cup at the basal end. This structure can introduce viscoelastic interaction with Deiters’ cells^29^, which serves as a basis for viscous coupling^17,30^. The present analysis does not show that viscous coupling is effective to enhance the amplitude of LO at high frequencies. Presumably OHCs are held elastically, at least in the basal high-frequency region of the cochlea.

Short hair cells are more effective, having larger values of amplifying parameter *u* because of their smaller values of $C_0$ and larger values of $k_o$. Indeed, basal cells, which operate high frequencies, are short. For example, basal cells in both guinea pigs and mice are short ($\lesssim 20\;\mu$m long), even though their length gradient is steeper for guinea pigs^31^.

The models examined in the present treatment assume that the main drag of the system is associated with HO. Such a condition could be realized if the main drag imposed on HO is outside the subtectorial gap.

However, the most likely source of the main drag of the system could be the shear in the subtectorial space between the tectorial membrane and the reticular lamina^32^. This picture indicates that the shear is associated with the motion of HO. If we accept that hair bundle bending is associated with this shear, the OHC should be stimulated by the displacement of HO. That leads to HO-stimulation models.

Can the hair bundle of the OHC be primarily stimulated by LO without incurring significant drag? Such a condition could be realized if LO moves the hair bundle in the direction perpendicular to the reticular lamina, resulting in effective bending of the hair bundle. However, the movement of LO should not incur significant drag. It would be possible that displaced water volume could be accommodated by local displacement of the TM owing to its pliability^33^ and mechanical anisotropy^34,35^. Then, the movement of LO does not result in viscous drag because it does not lead to fluid flow along the gap.

We assumed that prestin, the motile protein that drives OHCs, undergoes conformational transitions fast enough so that mechanical constraints determine the frequency dependence. This assumption is consistent with the experimental data on isometric force generation by OHC^36^ and current noise spectrum of OHC membrane^37^. It is also in line with a recent analysis that movement of organ of Corti measured with OCT is consistent with the cycle-by-cycle force application^38^.

Recent repots that the roll-off frequencies of the voltage-dependent component of OHC membrane capacitance suggest 30 kHz,^39^. This limiting frequency could reflect extracellular factors, such as viscoelastic process, of their experimental configuration. Given large gains obtained by coupled oscillators, this issue is not so important because the amplitude is attenuated by a factor $1/(1+\omega _g/\omega )$ for a given gating frequency $\omega _g$^40^. Even if the gating frequency is 20 kHz, this attenuation factor is 1/3 at 40 kHz, a small fraction of the expected gain.

Cochlear models that are proposed to explain the performance of the mammalian ear, particularly the tuning curve, can be classified into two groups: microscopic and macroscopic models.

Numerous recent theoretical models do assume multiple degrees of freedom^41^. These models are of particular interest in recent years because they can be related to recent OCT experiments. These models can, in principle, overcome the issue of input impedance by choosing suitable parameter values because of their models have multiple degrees of freedom.

Macroscopic models of cochlear mechanics^42^ also are of interest because they assume that OHCs apply force directly on the BM. Such models ignore issues resulting from microscopic structure, such as low-pass characteristics of intrinsic cellular electric circuit and impedance mismatch.

Interestingly, however, the present analysis of coupled oscillator models shows a resemblance to the assumptions of those macroscopic models: The amplifier gain is not seriously constrained by the impedance mismatch. The difference in the amplitude and in the phase of the two oscillators is rather small. This observation does show the viability of these macroscopic models as giving approximate descriptions. However, it may not indicate the physical details in the inner the ear.

## Conclusions

Counteracting viscous drag by the motile mechanism of OHCs is an important necessary condition for hearing in the mammalian ear.

A large impedance mismatch between the stiff BM and soft OHCs impedes energy transmission from the softer OHC to the oscillation of the stiffer BM if the BM and the OHC are in the same oscillator. However, if these elements are incorporated into separate oscillators that are coupled, a significant improvement in the efficiency of energy transmission can be achieved.

Among the modes of motion examined, the system of elastically coupled with LO-stimulated OHC is most effective in utilizing the OHC as the amplifier. Under optimal conditions, both amplitudes and phases of these oscillators are close because these oscillators are strongly coupled.

The models examined here are the simplest possible cases. The real ear would be much more complex and can have more modes of motion. Nonetheless, these simple model systems could provide some insight into the working of the real system. It is likely that multiple modes of motion supported by the complexity of the organ of Corti are essential for the performance of the mammalian ear by making OHCs effective as the amplifier.

## Methods

### Single mode of motion

**Fig. 5:**
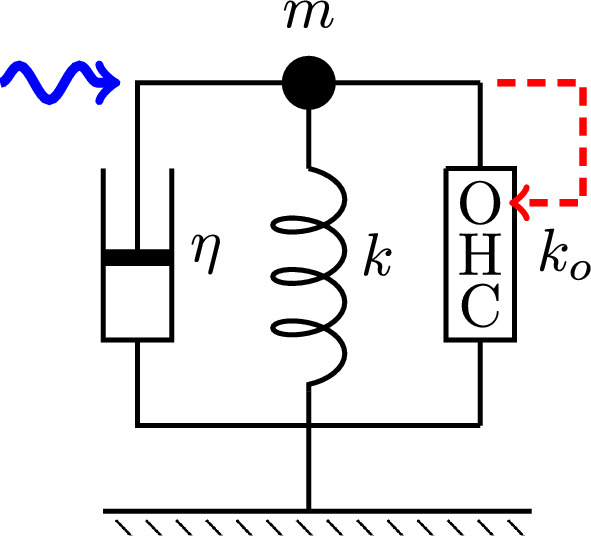
A single oscillator model. The oscillator consists of mass *m*, an external elastic element with stiffness *k*, a damper with drag coefficient $\eta$, and an outer hair cell (OHC), which responds to the movement of the mass (dashed red arrow). This system is driven by a sinusoidal waveform with angular frequency $\omega$ (wavy blue arrow).

Let us start from a simple model oscillator system, into which an OHC is incorporated (Fig. 5). The equation of motion can be formally written as

1 $$\begin{aligned} & \left( m\frac{d^2}{dt^2}+\eta \frac{d}{dt}+k_o+k \right) X=F_\textrm{OHC}+F, \end{aligned}$$

where *m* is the mass, $\eta$ drag coefficient, *k* the stiffness of the external elastic load, $k_o$ the material stiffness of the OHC, *X* is the length of the OHC, and $k_e$ the stiffness of external load. *F* is an external force and $F_\textrm{OHC}$ is the force generated by the OHC.

Consider the case, where a force *F* applied externally has a periodic waveform with angular frequency $\omega$. We can write

2a $$\begin{aligned} V&=V_0+v\exp [i\omega t],\end{aligned}$$

2b $$\begin{aligned} X&=X_0+x \exp [i\omega t], \end{aligned}$$

2c $$\begin{aligned} F&=F_0+f \exp [i\omega t], \end{aligned}$$

where *V* is the receptor potential, which is generated by the mechanosensitivity of the hair bundle.

In the frequency range, where capacitive conductance is greater than ionic conductance of the cell membrane and for a small amplitude *x*, the equation of motion can be expressed as^6^

3 $$\begin{aligned} x(-\overline{\omega }^2+i\overline{\omega }/\overline{\omega }_\eta +1+\gamma B)=i\frac{\gamma A}{\overline{\omega }} \hat{r}+(1+\alpha \gamma )\overline{f}, \end{aligned}$$

where $\overline{\omega }(=\omega /\omega _r)$ is the frequency normalized by the mechanical resonance frequency $\omega _r=\sqrt{(k+k_o)/m}$ and $\overline{\omega }_\eta$ is normalized viscoelastic roll-off frequency. The quantity $\gamma$ represents relative voltage sensitivity of prestin, the motile membrane protein of OHCs^43^. Its maximal value is set to unity (i.e. $0\le \gamma \le 1$) and it will be referred to as “the electromotile (em) parameter” or the “operating point parameter.”

The factor *A* is related to the force generation the response of the OHC through the mechanosensitivity of the hair bundle (HB) and $\alpha$ is a small factor ($<0.1$) due to the direct piezoelectric mechanosensitivity of the cell body^6^. For this reason, the resonance in which OHC is involved is semi-piezoelectric rather than pure piezoelectric^6^. For this reason, this factor can be ignored. The factor *B* represents the stiffness change associated with conformational changes of the motile element. Their definitions are given in Appendix A. The quantity $\overline{f}$ is given by

4 $$\begin{aligned} \overline{f}&=f/(k+k_o), \end{aligned}$$

The quantify $\hat{r}$ is the relative change of hair bundle resistance, which can be proportional to *x*. Thus, we may put $\hat{r}=g x$. This substitution leads to

5 $$\begin{aligned}&[-\overline{\omega }^2+i(\overline{\omega }/\overline{\omega }_\eta - \gamma gA/\overline{\omega })+1+\gamma B]x=\overline{f}. \end{aligned}$$

Notice that the factor *A* counteracts drag and factor *B* increases the stiffness of the cell due to strain-induced polarization^6^. Eq. 5 assumes that the mechanosensitivity of the piezoelectric cell body is smaller than that of the hair bundle, leading to minor underestimates of the amplitude (up to 10%)^6^. This assumption makes the connectivity, whether series or parallel, of the OHC unimportant and further facilitates the application of the present approach to coupled oscillator models.

The performance of the OHC in the system can be quantified by power gain $G(\omega )$, which is the ratio of power output to power input. Power output can be determined by the viscous dissipation $\eta \,|dX/dt|^2$ because elastic energy is recovered during a full cycle. Power input can be expressed as $\textrm{Re}[F\cdot dX/dt]$. Thus, power gain can be expressed by

6 $$\begin{aligned} G(\omega )&=\eta \omega ^2|x|^2/|\omega f\; \textrm{Im}[x] |\nonumber \\&=\eta \omega |x|/(f \sin \phi ), \end{aligned}$$

where *x* is given by Eq. 5, $\textrm{Re}[...]$ and $\textrm{Im}[...]$ represent, respectively, real part and imaginary part, and $\phi$ is the absolute value of the phase angle of *x* with respect to the external force. In the absence of the OHC, i.e. $A=B=0$, we obtain $G(\omega )=1$ as expected.

### Coupled oscillator models

**Fig. 6:**
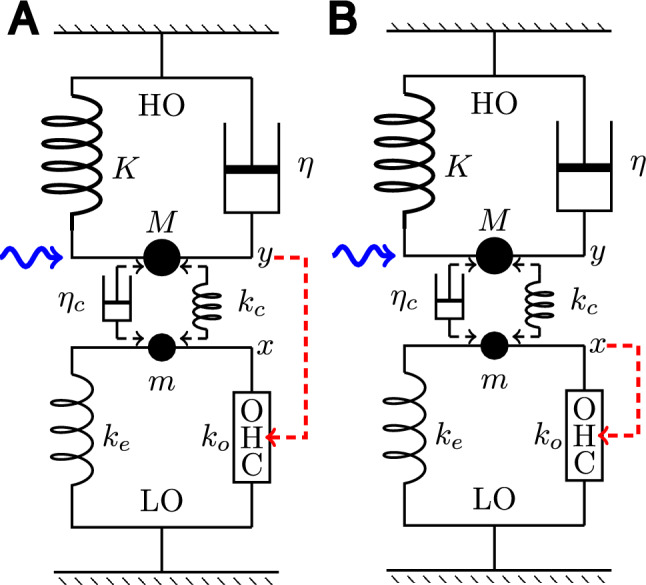
Coupled oscillators. (**A**) HO driven oscillator model, in which the OHC is stimulated by the motion of HO, and (**B**) LO driven oscillator model, in which the input to OHC comes from LO. HO (top) consists of mass *M*, an elastic element with stiffness *K*, and a damper with drag coefficient $\eta$. LO (bottom) consists of mass *m*, an elastic element with stiffness $k_e$, and an OHC, which responds to the movement of HO (dashed red arrow). HO is driven by a sinusoidal waveform with angular frequency $\omega$ (wavy blue arrow) and the two oscillators are coupled either by an elastic element with stiffness $k_c$ or by a damper with viscous coefficient $\eta _c$.

Consider a system with two harmonic oscillators, light and heavy. The light oscillator (LO) consists of an OHC and an elastic load $k_e$ and inertia *m*. The displacement of this oscillator is *X*. The heavy oscillator (HO), which includes the BM, consists inertia *M*, an elastic element with stiffness *K*, and viscous load with drag coefficient $\eta$. We assume, in general, that the two oscillators are coupled by an elastic element with stiffness $k_c$ or a viscous element with drag coefficient $\eta _c$. An external force *F* is applied to HO (Fig. 6). The motion of the two oscillators are described by

7a $$\begin{aligned} \left( m \frac{d^2}{dt^2}+\eta _1 \frac{d}{dt}+k_e+k_o \right) X&=F_\textrm{OHC}+\left( k_c+\eta _c\frac{d}{dt}\right) (Y-X)\end{aligned}$$

7b $$\begin{aligned} \left( M \frac{d^2}{dt^2}+\eta _2 \frac{d}{dt}+K\right) Y&=\left( k_c+\eta _c\frac{d}{dt}\right) (X-Y)+F(t), \end{aligned}$$

where $F_\textrm{OHC}$ is active force exerted by the OHC, which is stimulated at its hair bundle. In addition, we assume that the main drag $\eta$ belongs to HO. If we assume the opposite that the main drag belong to LO, the OHC cannot affect the oscillation of HO.

Classical analyses show that energy transfer between two coupled oscillators is rather complex despite the apparent simplicity of the equations^44,45^. Here we examine only the response to continuous sinusoidal stimulation.

8a $$\begin{aligned} F&=F_0+f _B\exp [i\omega t],\end{aligned}$$

8b $$\begin{aligned} X&=X_0+x \exp [i\omega t],\end{aligned}$$

8c $$\begin{aligned} Y&=Y_0+y \exp [i\omega t], \end{aligned}$$

where $\omega$ is the angular frequency of stimulation. Notice also that the notation $f_B$ is used for the amplitude of the external force in the systems of coupled oscillators. Now $F_\textrm{OHC}$ can be expressed in a manner similar to in Eq. 5 using *A* and *B* because the force directly applied to the cell body is only of secondary importance for force production as discussed in the single mode oscillator.

In the case of viscosity coupling these quantities are defined by Eq. 14. In the case of elastic coupling, *A* and *B* need to be replaced respectively by $A_e$ and $B_e$, where the stiffness ratios are replaced due to the presence of an additional elastic element. Namely, $k_o/(k_o+k_e)$ in *A* is replaced by $k_o/(k_o+k_e+k_c)$ in $A_e$ and $k_e/(k_e+k_o)$ in *B* by $(k_e+k_c)/(k_e+k_c+k_o)$ in $B_e$.

In general, bending of the OHC hair bundle that results in $\hat{r}$ may depend on *x*, the displacement of LO, as well as on *y*, the displacement of HO. For simplicity, two extreme cases will be examined here: $\hat{r}$ depends only on *y* (HO-driven mode), or only on *x* (LO-driven mode). Coupling may have both viscous and elastic components. However, we assume coupling is purely elastic or purely viscous for simplicity.

Power gain $G(\omega )$ is obtained analogous to Eq. 6 for the simple oscillator,

9 $$\begin{aligned} G(\omega )=\eta \omega |y|/(f_B \sin \varphi ), \end{aligned}$$

where $\varphi$ is the absolute value of the phase angle of *y*. Notice that amplifier gain here is determined by the amplitude *y* of HO because both external force and drag work on HO.

#### HO-driven case

We have assumed that the main drag of the system belongs to HO. If this drag is due to the shear in the subtectorial space, it may appear natural that HO motion stimulates the hair bundle of the OHC (Fig. 6 A), i.e. $\hat{r}=g Y$.

##### Viscous coupling

Let $\omega _\eta$ be the viscoelastic roll-off frequency of HO. With new parameters defined by $s=\!K/k$ and re-defined external force amplitude $f=f_B/K$, the set of equations can be written as

10a $$\begin{aligned}&[-(\overline{\omega }/\overline{\omega }_1)^2 +1+is\overline{\omega }/\overline{\omega }_c+\gamma B]x-i[\gamma Ag/\overline{\omega }+s\overline{\omega }/\overline{\omega }_c]y=0,\end{aligned}$$

10b $$\begin{aligned}&-i\overline{\omega }/\overline{\omega }_c \;x+[-\overline{\omega }^2+i\overline{\omega }/\overline{\omega }_\eta +1+i\overline{\omega }/\overline{\omega }_c]y=f, \end{aligned}$$

where $\omega _r$ is the resonance (angular) frequency of HO, i.e. $\omega _r^2=K/M$, and $\overline{\omega }_c=\omega _c/\omega _r$ with $\omega _c=K/\eta _c$. All frequencies are normalized to $\omega _r$. For example, $\overline{\omega }=\omega /\omega _r$. The quantity $\overline{\omega }_1$ is the ratio of the resonance frequency of the LO to that of HO. Notice that *f* has the dimensionality of length.

##### Elastic coupling

Coupling introduced by an elastic element $k_c$ would elevate the resonance frequency of LO more than that of HO. The parameter $\omega _1$ adjusts the resonance frequency of LO.

11a $$\begin{aligned}&[-(\overline{\omega }/\overline{\omega }_1)^2 +1+cs+\gamma B]x-[i\gamma Ag/\overline{\omega }+cs]y=0,\end{aligned}$$

11b $$\begin{aligned}&-cx+[-\overline{\omega }^2+i\overline{\omega }/\overline{\omega }_\eta +1+c]y=f, \end{aligned}$$

where *c* is defined by $c=\!k_c/K$.

#### LO-driven case

An alternative source of OHC stimulation is the displacement *x* of LO. This path of stimulation creates a direct feedback loop (Fig. 6 B).

##### Viscous coupling

The set of equations of motion can be written as

12a $$\begin{aligned}&[-(\overline{\omega }/\overline{\omega }_1)^2 -i\gamma Ag/\overline{\omega }+1+is\;\overline{\omega }/\overline{\omega }_c +\gamma B]x-i s\;\overline{\omega }/\overline{\omega }_c y=0,\end{aligned}$$

12b $$\begin{aligned}&-i\;\overline{\omega }/\overline{\omega }_c x+[-\overline{\omega }^2+i\overline{\omega }/\overline{\omega }_\eta +1+i\;\overline{\omega }/\overline{\omega }_c]y=f. \end{aligned}$$

The phase relationship between *x* and *y* is determined by Eq. 12a.

##### Elastic coupling

The time dependent components follow the following equation

13a $$\begin{aligned}&[-(\overline{\omega }/\overline{\omega }_1)^2 +1+cs+\gamma B-i\gamma Ag/\overline{\omega }]x-csy=0,\end{aligned}$$

13b $$\begin{aligned}&-cx+[-\overline{\omega }^2+i\overline{\omega }/\overline{\omega }_\eta +1+c]y=f, \end{aligned}$$

### Definitions of parameters

For the system that corresponds to Fig. 5, the factors *A*, *B*, and $\alpha$ are given by^6^

14a $$\begin{aligned} A&= u(i_0g-\sigma q/C_0)k_o/(k+k_o),\end{aligned}$$

14b $$\begin{aligned} B&=u_a k/(k+k_o)+u_q,\end{aligned}$$

14c $$\begin{aligned} \alpha&=u_a k/(k+k_o). \end{aligned}$$

Quantities *u*, $u_a$, and $u_q$ represent hair bundle sensitivity and piezoelectricity of the OHC. More specifically, *u* represents mechanoelectric coupling, $u_a$ mechanical term, and $u_q$ electrical term, while all of them depend on hair bundle sensitivity. See Table 1 for definitions.

The elastic load *k* depends on the configuration. For single mode oscillator (SO), $k=K$, for coupled oscillators, $k=k_e+k_c$, where for viscous coupling, $k_c=0$ (See Fig. 6).

## Parameter values

To find a set of adequate parameter values for a high frequency (40 kHz) location of guinea pigs, the parameter values are examined for a more readily obtainable 20 kHz location, and then they are extrapolated to 40 kHz location.

### Cellular factors (20 kHz)

For a 20 $\mu$m-long cell, typical of the 10–20 kHz cochlear region, we have for the linear capacitance $C_0 = 8$ pF and the maximal electromotile displacement $aN = 1 \mu$m, which is 5% of the resting cell length. Most in vitro experiments show the unitary motile charge of $q = 0.8 e$, where *e* is the electronic charge. The resting membrane potential is near the optimal range for the motile element. The resting basolateral resistance is 7 M$\Omega$ and the resting membrane potential of $-50$ mV requires the resting apical resistance of 30 M$\Omega$. These values lead to $i_0 = 4$ nA. These parameter values are summarized in Table 2.

**Table 2:** Parameter values at 20 kHz location.

Parameter	Definition	Value	Source
$C_0$	Structural capacitance of OHC	8 pF	
$k_o$	Structural stiffness of OHC	20 mN/m	^46^
$k_e$	Elastic load on OHC	Adjustable	
*K*	BM stiffness per OHC	200/3 mN/m	See text
$s_o$	Ratio $K/k_o$	10	
*s*	Stiffness ratio $K/(k_o+k_e)$	$\sim$5	
$i_0$	Mechanoreceptor current	4 nA	^47^
$\sigma$	Membrane conductance	40 nS	^48^
$\omega _\eta /\omega _r$	Viscous roll-off frequency	12.5	see text
*g*	Hair bundle sensitivity	1/(25 nm)	^49,50^
*q*	Motile charge	0.8 *e*	^39,46^
*a*	Prestin displacement	$6.3\times 10^{-5}$ nm	
*N*	Number of motile unit	$1.6\times 10^7$	[†]

[†] Corresponds to peak nonlinear capacitance of $\sim$17 pF

The stiffness $k_o$ of a 20 $\mu$m-long OHC is about 20 mN/m, given the specific stiffness of 510 nN/m per unit strain^46^. However, this value may have some uncertainty. The bottom part, up to 10 $\mu$m, of the OHC is held by the Deiters’ cup. If this structure works as a damper^30^, the length of elastic displacement is larger because the displacement includes the part within the cup. If, on the contrary, the structure is tight and rigid, not allowing slippage, the value of $k_o$ must be higher.

The parameter values in Table 2 lead to a set of values for an OHC at 20kHz location:

15 $$\begin{aligned}&gi_0u(20k)=1.4, \quad u_a(20k)=0.08, \quad u_q(20k)=2. \end{aligned}$$

The second term containing $\sigma$ in the coefficient *A* is not significant at 20 kHz location because the ratio $i_o/\sigma$ is small unlike at 4 kHz location^6^. That is because HB conductance, which primarily determines $i_0$, increases much sharper than the cellular conductance toward the base. The coefficient *B* is approximately 2. It is not sensitive to the ratio of the load ratio because it $u_a$ is smaller than $u_q$.

### Cochlear factors (20 kHz)

Now assume that the BM is the source of the stiffness *K*. The best frequency location for 20 kHz in guinea pigs is 3 mm from the stapes according to the Greenwood function, assuming the length of the BM is 18.5 mm^51,52^. The stiffness of the BM at this location is about 0.21 N/m measured with a probe with 25 $\mu$m diameter tip^53^. This value is compatible with the ones obtained with a probe with 10 $\mu$m tip^54^. Thus, the stiffness ratio $s_o$ is $\sim$10.

The intrinsic mechanical resonance frequency of the location is somewhat uncertain because of the so-called “half-octave shift”^55^. If the intrinsic mechanical resonance corresponds to the “passive” condition, the resonance frequency of the 20 kHz location is 14 kHz ($\omega _r=20/\sqrt{2}$) being a half octave lower. However, it could be the opposite because viscous damping brings the peak to a lower frequency if it is not counteracted.

A significant contribution to friction is expected from the gap between the tectorial membrane and the reticular lamina. The friction coefficient of this gap can be estimated by a formula $\mu S^2/d$, where $\mu$ is the viscosity of the fluid, *S* the area of the gap per OHC, and *d* the gap, provided that the thickness of the boundary layer, which is $\sim 3.6\; \mu$m for 20 kHz^56^, is greater than the gap. If we assume *S* is $10\mu m\times 15\mu m$ and *d* $1\mu m$, the friction coefficient is $1.2\times 10^{-7}$ N/m^5,8^. With the resonance frequency of 14 kHz, the gap friction leads to a value 12.5 for $\omega _\eta /\omega _r$.

### Extrapolation to higher frequencies

Of the OHC parameters, *B* does not depend heavily on the resonance frequency because it is dominated by the electrical term $u_q$ (See Eq. 14b). The cellular component $gi_0u$ of the amplification parameter *A* decreases at higher frequency locations because *u* is inversely proportional to the resonance frequency $\omega _r$. However, such a reduction can be compensated by other factors: OHCs at higher frequency locations have larger resting current $i_0$ owing to larger hair bundle conductance and larger structural stiffness $k_o$ of the cell body owing to its shorter cell length (See Eq. 14a and Table 1).

At higher frequency locations, the stiffness ratio *s* is expected to increase because the stiffness of the BM would increase more than the stiffness of OHCs. However, we proceed by assuming that the ratio *s* remains the same. The reason is an uncertainty in the effective length of OHCs. The stiffness is inversely proportional to the effective length of the lateral membrane, which is harder to determine with shorter OHCs because the connectivity in Deiters’ cup is ambiguous as described earlier. In addition, somewhat higher values for *s* do not lead to significantly different results. For those reasons, the values for $gi_0u$ at the 40 kHz location is likely somewhat higher than a half of the value at the 20kHz location, expected from the difference in the resonance frequency $\omega _r$.

To examine high frequency performance aiming at 40 kHz, the upper limit of the auditory frequency range for guinea pigs, a numerical analysis is performed, somewhat underestimating the effectiveness of OHCs by assuming the following set of parameter vales:

For a single oscillator,

$$\begin{aligned} A=0.7 \; k_o/(k_o+K), \quad B=2, \\ K/k_o=10, \quad \overline{\omega }_\eta =10, \end{aligned}$$

where *K* is the stiffness of the basilar membrane.

For coupled oscillators,

$$\begin{aligned} A=0.7\; k_o/(k_o+k_e+k_c), \quad B=2, \\ K/k_o=10, \quad k_o=k_e, \quad \overline{\omega }_\eta =10, \end{aligned}$$

where $k_e$ is the stiffness of the direct elastic load to OHC and $k_c$ the stiffness of the elastic coupling element. For viscous coupling, $k_c=0$. Adjustable parameters are $\overline{\omega }_1$ and a coupling parameter, either $c=k_c/K$ or $\overline{\omega }_c=K/(\eta _c\omega _r)$ (See Fig. 6).

## Supplementary Information

Below is the link to the electronic supplementary material.

Supplementary Material 1

## Data Availability

All data generated or analyzed during this study are included in this published article.
