# Supplementary material for: Coupled oscillator mechanism extends the amplifying effect of outer hair cells to high frequencies
Source: Sci Rep. 2025 Dec 22;15:45122. doi: 10.1038/s41598-025-33079-3 (PMC12748779; doi:10.1038/s41598-025-33079-3)
Supplement: Supplementary file 1 — Supplementary Material 1 [file 41598_2025_33079_MOESM1_ESM.pdf]

Supplementary Material

Here viscosity coupled oscillators are presented. Each example is obtained after attempts of optimizing the amplitude gain. These cases do show amplification, but not as large as elasticity coupled cases.

Viscosity coupled HO-driven case

The Deiters' cup that links an OHC with the BM via Deiters' cell could provide viscous coupling due to its morphology. For this reason, it is of interest to examine the effect of viscous coupling.

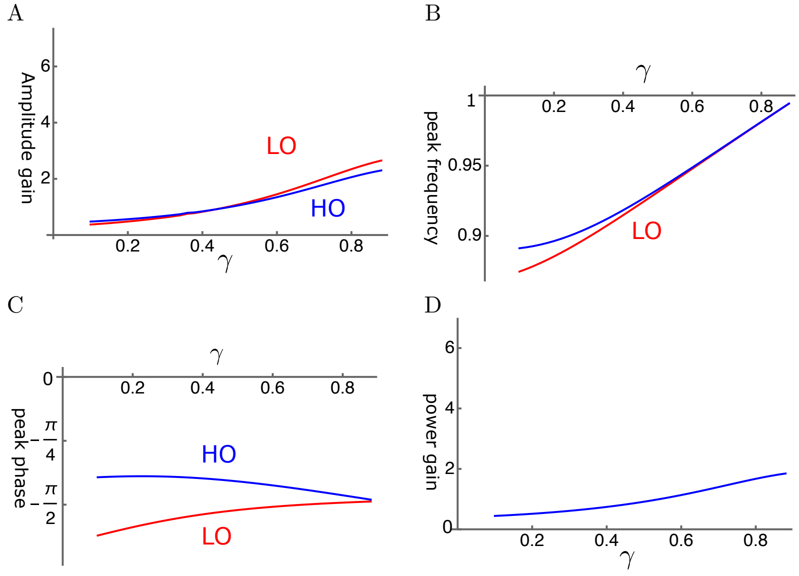

Figure S1: HO-driven viscosity coupled oscillators (HOV). A: Amplitude gain over SO mode, B: Peak frequency, and C: Peak phase with respect to the external force of each oscillator are plotted against γ, the operating point variable. LO (red) and HO (blue). D: Power gain is closely associated to HO amplitude. The set of parameter values: $1/\overline{\omega}_c$=0.3, ω_1=0.33 in Eq. 10.

Even though viscous coupling of the oscillators can have an amplifying effect, it does not appear to be effective. Amplitude gain tends to be small (Fig. S1A). Peak frequency of LO and that of HO are close to each other except for at small values of γ (Fig. S1B). The phase of HO is ahead of LO (Fig. S1C). Since the force LO applies to HO is by $\pi/2$ ahead of the phase of LO, LO amplifies the motion of HO. Power gain is rather small (Fig. S1D) as expected from the amplitude of HO.

Viscosity coupled LO-driven case

The amplitude gain for both LO and HO increases with the operating point variable γ (Fig. S2A). HO has somewhat higher peak frequency than LO for small γ (Fig. S2B). The phases of HO and LO are both close to $\pi/4$ and HO tends to be ahead of LO except for where γ is small (Fig. S2C). The power gain is not very large.

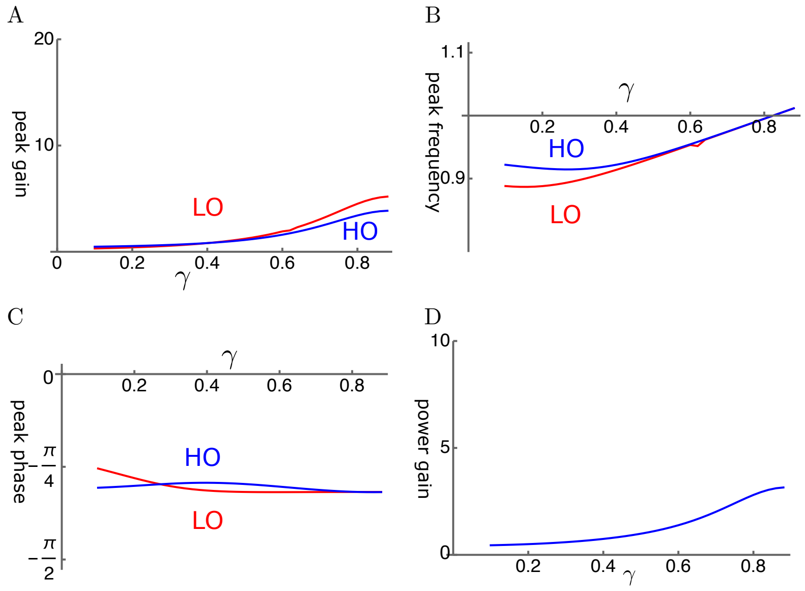

Figure S2: LO-driven viscosity-coupled oscillator (LOV). A: Amplitude gain over SO mode, B: Peak frequency, and C: The phase with respect to the external force, of each oscillator are plotted against γ. LO (red) and HO (blue). $\overline{\eta}_c = \eta_c \omega_r / K$. D: Power gain. The set of parameter values: $1/\overline{\omega}_c$=0.2, ω_1=0.36 in Eq. 12.
